# Water vapor thermal therapy to alleviate catheter-dependent urinary retention secondary to benign prostatic hyperplasia

**DOI:** 10.1038/s41391-019-0187-5

**Published:** 2019-11-18

**Authors:** Kevin T. McVary, Bradley Holland, J. Randolf Beahrs

**Affiliations:** 1 Department of Urology, Stritch School of Medicine, Loyola University Medical Center, Maywood, IL USA; 2 Division of Urology, Southern Illinois University School of Medicine, Springfield, IL USA; 3 Minnesota Urology, Woodbury, MN USA

**Keywords:** Prostatic diseases, Outcomes research

## Abstract

**Background:**

Water vapor thermal therapy utilizes convectively delivered thermal energy to target ablation of obstructive prostatic tissue. We report results of this thermal therapy for relief of nonneurogenic complete urinary retention associated with BPH.

**Patients and methods:**

We conducted a retrospective analysis of 38 catheter-dependent men with complete urinary retention consecutively enrolled in a registry in two centers: median age 75.5 years and multiple comorbidities, median prostate volume 58.5 cc (23–153), median 2 failed trials without catheter (TWOCs), and median catheter dependency 3 months (0.3–35). The Rezūm™ System thermal therapy procedure was performed in an ambulatory surgery center with conscious sedation or an office procedure room with a modified periprostatic block. Water vapor injections were customized to the configuration of the hyperplastic gland, including median lobe and/or enlarged central zone.

**Results:**

Of the 38 treated patients, one was lost to follow-up and 26 of 37 (70.3%) voided spontaneously (mean of 1.6 ± 0.8 TWOCs) and were catheter free a median of 26 days (range 4–65) after the procedure; 18 of these 26 (69%) patients discontinued BPH medications. No significant differences in age, prostate volume, number of water vapor injections, or presence of the median lobe were associated with predicting a successful treatment outcome. Duration of follow-up for 20 catheter-free patients was a median of 475 days or 15.8 months (140–804 days); six patients were followed a median of 31.5 days (0–60). Adverse events were infrequent, mild, and resolved quickly including dysuria in five patients (13%), gross hematuria in four (10.5%), and UTIs in two (2.6%) with indwelling catheters.

**Conclusions:**

Water vapor thermal therapy may provide an effective and safe alternative to surgical treatment in this group of catheter-dependent patients in complete urinary retention.

## Introduction

Water vapor thermal therapy with the Rezūm™ System utilizes convectively delivered thermal energy to target ablation of obstructive prostatic tissue. This minimally invasive surgical treatment now has a substantial history as an efficient, effective, and safe modality to treat clinically significant BPH [1–4]. The procedure is currently recommended for ablation of lateral and median lobes of the prostate [5]. It may be performed in an outpatient or office-based setting without general anesthesia and provides durable relief of LUTS. It may be offered to patients as first-line treatment for moderate-to-severe LUTS due to BPH and appears to lower the rate of clinical progression of BPH while preserving sexual function compared with daily long-term use of pharmaceutical agents [6]. This thermal therapy can be considered as an alternative before or after pharmacotherapy, for patients reluctant to undergo a traditional surgical procedure, and for those at high anesthesia risk due to poor health and comorbidities.

Urinary retention is a major risk factor associated with BPH, which is in part responsible for domains of LUTS including hesitancy, intermittency, poor flow, and large PVRs. The natural history of LUTS/BPH may culminate in urinary retention, an adverse event men rank as significant as myocardial infarction or cerebral vascular accident [7]. Catheter-dependent patients seek treatment to improve bladder function and quality of life, particularly to reduce long-term catheter complications. Operative procedures to relieve bladder outlet obstruction from BPH in older or frail patients with high comorbidities are fraught with potential complications including anesthesia risk, UTI with septicemia, surgical bleeding, urethral stricture, and possible incontinence [8].

The aim of this report is to share our experience with water vapor thermal therapy for treatment of medically refractory, complete urinary retention to achieve successful cessation of catheter dependency in older patients, most considered unsuitable for traditional bladder outlet surgery.

## Patients and methods

### Study procedures

Patients were consecutively evaluated at two urology centers, a university clinic, and a private urology practice, and compiled into a single retrospective registry—Rezūm Retention Registry. Accrual was typically by referral after initial presentation in an emergency department (13), by a primary care physician or urologist (12), or established and new patients in the private practice (13). Once accrued, patients were evaluated with history, physical examination, and transrectal ultrasound prostate volume. They were treated one time with the water vapor thermal therapy procedure. To avoid confusion with urinary retention terminology specifically for these patients who had variable durations of retention, they were classified as being in complete urinary retention. The primary objective of the water vapor thermal therapy procedure was to achieve patients being able to void without further intervention and be relieved of catheter dependence. A secondary objective was discontinuance of BPH medications. Institutional review board approval was obtained; patient confidentiality was strictly maintained for the retrospective review.

### Thermal therapy procedure

Convective radiofrequency water vapor thermal therapy with the Rezūm™ System (Boston Scientific Corporation, Marlborough, MA) utilizes transurethral endoscopic guidance. The intent of the procedure is to create continuous, overlapping ablative lesions running parallel to the natural slope of the prostatic urethra, eliminating tissue interfering with natural function. The technology, device, and procedure have been previously described in detail [1, 3, 9]. Briefly, contours of the prostate and planned disbursement of thermal lesions are determined at cystoscopy. The total number of vapor treatments in each prostate lobe is determined by length of the prostatic urethra and can be customized to the configuration of the hyperplastic gland, which may include median lobe or enlarged central zone. For patients who undergo a Rezūm procedure, an indwelling catheter may be left in place for several days to allow for reduction in edema associated with ablated tissue and to avoid irritative symptoms when the patient is able to void spontaneously. The expectations and guidance for postoperative management after the thermal therapy procedure have been reported [9].

### Statistical methods

Descriptive statistics were used to describe baseline and follow-up values for all variables. Data are presented as the mean ± SD or median. As the response groups were small and unequal in size the two-sample Wilcoxon test was used to assess whether there were differences between those who achieved catheter independence after treatment and those who did not. *P* < 0.05 was considered statistically significant.

## Results

This Rezūm Retention Registry retrospective analysis included 38 patients in urinary retention associated with BPH and dependent on either an indwelling catheter or clean intermittent self-catheterization (CIC). These patients were unable to empty their bladder in any sufficient way. Most patients were in poor health or unsuitable for surgery owing to existing cardiovascular, pulmonary, gastroenterological, neurological, or other diseases and for whom a surgical alternative was needed (Table 1). The university center described 13 of 18 (72%) patients as high-risk chronic urinary retention (CUR) due to concomitant hydronephrosis, recurrent UTIs, urosepsis, ≥stage 3 chronic renal disease, or glomerular filtration rate <60 mL/min. This risk assessment for CUR was not documented at the private urology practice. Patient median age was 75.5 years (range 59–90); median prostate volume was 58.5 cc (range 23–153).

**Table 1:** Baseline characteristics and outcomes after water vapor thermal therapy

	All patients* N = 38	Catheter free *N* = 26	Catheter dependent *N* = 11	P*
**Baseline characteristics **				
Age, years, mean	76.0 ± 9.1	75.0 (9.7)	79.2 (7.2)	0.33
Median [range]	75.5 (59–90)	75 [59–89]	76 [70–90]	
Prostate size, cm^3^	64.4 ± 35.4	62.3 (32.2)	72.1 (45.4)	0.77
Median [range]	58.5 (23–153)	52 [23–137]	61 [25–153]	
Prior bladder function assessment	4	3	1	
Using anticoagulant medications	9	5	4	
ASA physical status classification				
ASA II	4 of 18	3	1	
ASA III	7 of 18	4	3	
ASA IV	7 of 18	6	1	
ASA not assessed	20	13	6	
Comorbidities reported^†^ (no. of patients)				
Cardiovascular	39 (24)	29 (17)	10 (7)	
Gastrointestinal	20 (18)	18 (16)	2 (2)	
Genitourinary	13 (11)	11 (9)	2 (2)	
Endocrinological	13 (11)	10 (9)	3 (2)	
Oncological	12 (8)	11 (7)	1 (1)	
Neurological	10 (6)	6 (4)	4 (2)	
Nephrological	5 (5)	2 (2)	3 (3)	
Pulmonary	3 (3)	3 (3)	0 (0)	
Duration of catheter dependence	4.5 ± 6.5	3.1 (2.1)	7.9 (11.2)	0.60
Months, Median [range]	2.5 (0.5–35)	2 [<1–8]	4 [<0.5–35]	
No. of previous failed TWOC			> 2	0.18
Median [range]	2 [0–4]	2.5 [1–4]	(Many on CIC)	
**Treatment and outcomes**				
No. of water vapor injections, mean [range]				
Total all zones treated	–	5.7 [2–10]	5.4 [2–10]	
Right and left lobe	–	4.6 [2–9]	4.1 [2–8]	
Median lobe	–	1.1 [0–3]	1.3 [0–2]	
No. with median lobe identified and treated (%)	–	19 of 25 (76.0%)	9 of 11 (81.8%)	1.00
Catheter type post procedure until TWOC, (*n*)	–	IDC (12) CIC (14)	IDC (6) CIC (5)	
Time to successful TWOC, days	–	26.6 (14.1)	–	
Median [range]		26 [4–65]		
Discontinued BPH medications, *n* (%)	–			
Yes		18/26 (69.2 %)	–	
No		5/26 (19.2%)	–	
Tapering until lost to follow up		1/26 (3.8%)	–	
Never used medications		2/26 (7.7%)	–	
Duration of follow-up for catheter-free patients (*n*) median, days, months [range]				
≤60 days (6)		31.5 days	–	
		[14–60 days]		
>61 days (20)		475 days or 15.8 months	–	
		[140–804 days]		

*One patient lost to follow up for outcome assessment

^†^Some patients had 2–4 comorbidities in one or more systems

Some patients were without comprehensive baseline information such as duration of LUTS/BPH, number of failed TWOCs, and assessment of bladder function; urodynamic studies were either not performed or results were not accessible. Although all patients had LUTS/BPH, a temporal history was documented in only 18 of 38 with a median duration of 3.5 years (range > 1–15). Catheter dependency before the thermal therapy procedure was a median of 3 months (range 0.3–35). Some patients had either long-term Foley catheter drainage or long-term CIC. For those patients (25 of 38) with information on number of TWOCs, the median was two failed TWOCs. Not all patients had a PVR in our record for various reasons, namely, sometimes the referral sources did not have means to measure PVR prior to insertion of catheter or patients had multiple failed TWOCs each with a variable PVR prior to reinsertion of the Foley catheter. There was no set protocol to declare the cardinal PVR. For 21 of 38 (55%) patients with a reported baseline PVR, median was 320 mL (range 100–2500).

All thermal therapy procedures were successfully performed in an ambulatory surgery center or office procedure room and completed without perioperative device or procedure-related AEs. A total of 14 of 18 (78%) university center patients were characterized as ASA class III or IV. These patients received conscious sedation during the thermal therapy procedure. The private practice performed a modified periprostatic block for anesthesia in all procedures as described previously [9]. After the thermal therapy procedure either an indwelling catheter was inserted or CIC was taught to the patient, depending on the patient’s willingness, ability to comply, and physical health. A TWOC was generally performed at 1 week and then weekly thereafter. This was done by a trial of “fill and void” checking the PVR thereafter once or 1–2 times weekly for 1 month, then at 3 months and 6 months.

Eighteen patients had an indwelling catheter and 19 patients performed CIC after the thermal therapy procedure before the voiding trials (Table 1). Twenty-six of the 37 evaluable treated patients were able to spontaneously void after the procedure for an overall success rate of 70.3% (Table 1). Only one patient was lost to follow-up immediately after treatment and remained catheter dependent at that time. Both clinical centers reported similar success rates, notably 13 of 18 (72.2%) and 13 of 20 (65%). Patients voiding spontaneously had a mean of 1.6 ± 0.8 TWOCs and were catheter free a median of 26 days (range 4–65) after thermal therapy. A total of 18 of 26 (69.2%) catheter-free patients were able to discontinue their oral BPH medications. While the factors for a successful or failed outcome could not be definitively determined, they did not appear to be related to any significant differences in age (*p* = 0.33), prostate volume (*p* = 0.77), or number of water vapor injections, or presence of the median lobe. The percentage of men with large prostates ≥80 cc was greater in those with TWOC successes (7 of 26, 28.0%; volume range 81–153 cc) versus TWOC failures (2 of 11, 22.2%; volume range 91–129 cc). The duration of time patients were catheter dependent before treatment, a median of 3 months (range 10 days to 35 months) was markedly variable. Duration of follow-up for 20 of 26 catheter-free patients was a median of 475 days or 15.8 months (range 140–804 days) and a relatively brief follow-up duration for six patients was a median of 31.5 days (range 0–60). With the exception of one patient who required catheter use almost 2 years after the thermal therapy procedure, the others were catheter independent at the last documented visit.

Nonserious anticipated adverse events that may develop after a rigid cystoscopic procedure were infrequent, of short duration and mild in severity. These events included dysuria in five patients (13%), gross hematuria in four patients (10.5%), and UTIs in two patients with an indwelling catheter post treatment (2.6%).

## Discussion

The current urologic state of the art management of BPH is evolving on a reasonably rapid scale. Minimally invasive procedures, easily administered in the office, appear to eclipse the need for aggressive pharmacologic management in most cases and avoid the cost and adverse effects of the drug therapy. Some BPH patients progress to a point of complete urinary retention either by significant prostate growth, underactive bladder contractility, or combination of both. This urinary retention is often the tipping point requiring surgical therapy with aggressive removal of obstructing tissue. Our observational study of several such patients demonstrates the advantages and acceptable outcomes of water vapor thermal therapy to reduce BPH burden.

Urinary retention remains a common indication for a TURP. Patients who present in retention due to high failure rates of medical management are typically offered TURP to free them from long-term catheter use. The success rate of TURP appears to be significantly affected by age. In one report of 95 men with mean follow-up of 704 days, 87.4% were catheter free either at the time of follow-up or prior to death. The mean age of catheter-free men was 74.3 years compared with 84.9 years for men who remained catheter dependent [10]. Patients operated on after complete urinary retention have an increased mortality rate, unrelated to retention, but reflecting the advanced stage of BPH usually seen in elderly patients presenting with multiple medical conditions.

The incidence and prevalence of CUR is unknown. However, publications from the United Kingdom have reported the incidence of CUR in men who underwent TURP ranging from 14 to 37% [11–13]. Due to the increased operative risk of TURP, as well as AEs for these patients, nonsurgical treatment alternatives are required to meet their needs. Studies in the early 2000s and after reported the first successes with TUMT for urinary retention with TWOC success rates ranging from 72 to 82% [14–19]. In a later randomized, controlled trial of catheter-dependent patients treated with TUMT or TURP/enucleation at 6 months of follow-up 79 and 88% of men were catheter free, respectively [18]. A high rate of UTIs occurred with both treatments, 33% for TUMT and 22% for TURP, however with a significantly greater number of serious AEs of 8.5% with TURP versus 1.6% for TUMT. A lower frequency of AEs for TUMT, 2% versus 17% for TURP, was also reported in patients with clinical BPH although without retention [20]. When comparing rates of restored spontaneous voiding in patients treated with TUMT and Rezūm, Rezūm patients in this study were older, their prostate volumes were greater, a higher percentage was identified as unsuitable for traditional surgery, and additionally there was a high rate of postoperative BPH medication cessation, an observation not reported in other studies. The convective water vapor thermotherapy in comparison to conductive TUMT ablation may represent a more efficient modality, which affords the ability to safely treat a hyperplastic central zone and median lobe.

There was great variability in pretreatment PVR and duration of catheter dependence for our study patients with recalcitrant urinary retention. Some men did not meet the consensus definition of nonneurogenic CUR supported by the AUA and International Continence Society [21, 22]. That is, CUR defined as an elevated PVR of ≥300 mL that persisted for at least 6 months and documented on two or more separate occasions. The evidence for this definition is considered expert opinion only as few urologists would recommend a 6 month delay before attempting an effective treatment for retention just to meet the time component therein [21, 23]. Clearly there is little standardization in the duration or PVR necessary for diagnosis and treatment of CUR. Research studies often use a PVR >300 mL to diagnose CUR; others have used 100, 400, and 500 mL [24]. In addition, urinary retention is produced from variable pathogenic mechanisms that create neurologic detrusor underactivity and/or chronic bladder outlet obstruction. Definitions aside, patients assessed herein may be typical of those with catheter-dependent retention presenting in community primary or urology practices or referred to tertiary university centers, many without previous or inconsistent medical care for their BPH and without prior full work-ups. The management of their condition must transcend definition and provide timely relief.

Although the study sample size is small the similar success rates between the two centers support the generalizability of the water vapor thermal therapy procedure in treating this recalcitrant high-risk group presenting with complete retention. Despite the solace found in ~70.3% TWOC success rate in effectively treating this population, we agree that further study using an active comparator group (i.e., water vapor thermal therapy versus TURP) and a standardized urodynamic assessment is needed. How to interpret the observation that successful TWOC was noted in those with larger prostates seems counterintuitive. One possibility is that the larger prostate cohort may represent those with more obstruction-linked retention rather than detrusor underactivity related retention. Without baseline urodynamics this putative dichotomy remains conjecture.

A limitation of this registry study includes the absence of thorough baseline assessments in some patients that would assist evaluation of comparative changes in urinary symptom scores and quality of life measures (i.e., I-PSS). In addition, in men whose retention has lasted many months prior to effective thermal therapy treatment, the validity of a recalled I-PSS score is known to be inaccurate muting the impact of a lack of baseline I-PSS in this cohort [25]. While these baseline evaluations are expected in a clinical trial, our observations with limited data may represent the reality in the clinical setting. This report lacks a uniform approach to the withdrawal of BPH/LUTS medications, but there is ample evidence herein that this is frequently possible.

Our reported results, outside the realm of a formal clinical study, support a nonsurgical and efficient therapeutic option with water vapor thermal therapy for patients presenting with catheter-dependent urinary retention. For elderly patients or those in poor physical health considered at high risk for intraoperative complications and postoperative morbidity, this study shows that for many of these patients water vapor thermal therapy could contribute considerably providing relief of their catheter and BPH medications and improving quality of life. Longer term follow-up to determine duration of catheter independence will be essential to evaluate merits of the Rezūm procedure.

## Conclusions

Water vapor thermal therapy was effective to restore successful spontaneous voiding in a majority of patients with obstructive BPH necessitating a urethral catheter for complete urinary retention. The water vapor thermal therapy represents a new technological approach for ablation of obstructive benign prostate adenomas. As a minimally invasive surgical procedure it represents an alternative for treatment of catheter-dependent urinary retention in elderly and frail patients who are at anesthesia risk for invasive surgical approaches to relieve retention.
